# Supplementary material for: Weak acid and pepsin reflux induce laryngopharyngeal mucosal barrier injury: A rabbit-model-based study
Source: PLoS One. 2025 Jan 24;20(1):e0315083. doi: 10.1371/journal.pone.0315083 (PMC11759997; doi:10.1371/journal.pone.0315083)
Supplement: Attachment — Submitted filename: REVIEWERS RESPONSE.pdf [file pone.0315083.s002.pdf]

I would like to thank the authors for their effort in this study. There are many serious concerns regarding this manuscript as highlighted below:

Title

I do not see what is special about this topic, "Weak acid and pepsin reflux induced laryngopharyngeal mucosal barrier injury: a study based on rabbit model Study LPRD on rabbit model."

1. Weak acid is generally believed to be gastric acid. Why are the authors avoiding writing the proper term? They should be specific! The weak acid could be anything.

2. It is established knowledge that LPRD causes reflux. What is so special about this study? The significance of the study is not properly documented.

3. The mention of "Study LPRD on rabbit model" is unnecessary.

Abstract

The method is not detailed! Sprayed with different concentrations of acid— which acid? Hydrochloric or sulfuric?

The tissues were observed with Hematoxylin and eosin. For what? What was the observer looking for? This is not informative at all!

The conclusion part is not understood and rather confuses the readers, especially the statement "This study found that acid, weak acid, and pepsin…"

Introduction

Why is mentioning weak acid instead of gastric acid important in this context?

The two statements here, "Compared with the mucosa of the esophagus, the mucosa of the throat lacks a resistance mechanism to gastric acid and is more susceptible to reflux injury. Acid and pepsin disrupt barrier function and epithelial permeability by perturbing junctional complexes," lack references.

The statement "Gill et al. [6] studied 18 patients diagnosed with LPRD and found that the expression of E-cadherin in the laryngeal mucosal epithelium was significantly reduced, and pepsin appeared" is not understood with the ending words "pepsin appeared."

The following sentence should have been broken into two for clarity.

The statement “Notably, as a result of genetic diversity, individual expression of calcium mucin protection mechanisms must be differences” is poorly written and not scientifically constructed.

The sentence “Therefore, the existence of throat mucosa damage is defined, and the diagnosis of LPRD is unreliable” is not clear. Who defined it, or what does the author mean here?

The next statement, “Therefore, we may need to look for objective diagnostic indicators that can reflect mucosal injury,” is poorly written. Also, the knowledge gap here is different from what was stated in the abstract: “In this study, rabbit models were used to simulate the response of the laryngopharynx under the synergistic effects of various acidic reflux environments and pepsin to reveal the response mechanism of weak acid reflux…”

Therefore, the authors sound inconsistent with their objectives!

Methodology

The methodology was poorly described. For instance, the statement “The animals were randomly assigned into six groups (five animals per group). During the 8-week study, the rabbits in each group were treated with pH 3, 5, 7, and normal saline solution as the matrix, adding or not adding pepsin at 1 mL/kg for 3 min of slow spraying, twice per day, through the special catheter” is unclear.

Which group received what? This was poorly described. No one understands what was done to the animals and how each group was treated.

Also, the phrase in this statement “each group were treated with pH 3, 5, 7” is ambiguous and confusing.

In the statement “After intramuscular anesthesia, the animals received laryngoscopy. Because there was no previous assessment of normal values for rabbits, we referenced the method reported by Zhang et al. and Lou et al.[8,9] using a standard RFS system described by Belafsky et al.[10] to analyze images,” what does this mean? The author did not provide a detailed explanation of what those references mean. Are they not referring to rabbits? Are they pointing to abnormal values?

Also, the authors just jumped to “What is instantaneous impedance?” They failed to provide some important background information in the introductory section. They just jumped to the methodology without detailed information. Overall, the authors sound non-communicating and unscientific in their writing form.

The statement “After 8 weeks, the animals were sacrificed by air embolism…” Why air embolism? What does this suggest? There was no detailed explanation of how this impacts the results.

Also, what does lanthanum nitrate do? What is DIS? What do the immunostainers reveal, and what are those immunomarkers under the section “2.3 HISTOPATHOLOGY”? Why only one immune marker to proof this concept of degradation? The information provided is below expectations without any details.

And so on….

Overall, the manuscript lacks clarity, proper documentation, and scientific rigor in its writing. The authors need to provide detailed explanations, proper references, and clear descriptions of their methodology and findings to make the study comprehensible and valuable.
