# Supplementary material for: Weak acid and pepsin reflux induce laryngopharyngeal mucosal barrier injury: A rabbit-model-based study
Source: PLoS One. 2025 Jan 24;20(1):e0315083. doi: 10.1371/journal.pone.0315083 (PMC11759997; doi:10.1371/journal.pone.0315083)
Supplement: Attachment — Submitted filename: Response to Reviewers.docx [file pone.0315083.s003.docx]

**Response to Reviewers**

**Reviewer #1**

Dear Dr. Arwa Flemban,

Thank you very much for your detailed and constructive feedback on our manuscript entitled " Weak acid and pepsin reflux induce laryngopharyngeal mucosal barrier injury: a rabbit-model-based study". We appreciate the time and effort you have dedicated to evaluating our work, here is my response.

1- Enhancement of English Punctuation and Style:

The manuscript has been revised by the professional editing service: Editage (www.edita ge.cn).

2- E-Cadherin Expression:

Regarding E-Cadherin expression, we have updated the images with higher resolution and included references to E-Cadherin and cancer-related progression in the discussion section.

3- Consider evaluating additional markers related to cancer development or gene mutations, as they could provide more conclusive evidence.

We have also supplemented the discussion with relevant literature evidence on the link between inflammation and carcinogenesis. Due to the limited availability of rabbit-related antibodies, we are planning to search for more suitable markers for our future research.

4- Inflammatory Markers:

Added discussion on the inflammatory response section.

5- Effect of Catheter and Standardization:

To ensure the reliability of our experimental results, we used a blank control group (normal saline group) to account for any influence of the catheter. Additionally, we selected non-catheterized tissues to minimize any potential effects.

6- Treatment Dosage:

In our study, we applied different concentrations of solvents to treat 1 mL/kg of rabbit throat for 3 minutes using slow spraying.

7- Expand on Pepsin's Role:

We have discussed the mechanism of pepsin on cell damage and its potential impact on the development of laryngeal carcinoma.

8- Improve Figure Legends:

we have made modifications to part of the legend.

Best regards,

**Reviewer #2**

Dear Dr. Kamoru Adedokun,

Thank you for your insightful comments and suggestions on our manuscript titled " Weak acid and pepsin reflux induce laryngopharyngeal mucosal barrier injury: a rabbit-model-based study". We appreciate the time and effort you have invested in providing feedback. Below is my response.

1. Title

This study focuses on examining the effect of weak acid and pepsin on the laryngeal injury. Existing research has focused more on damage to the throat mucosa caused by strong acids. PPI treatment for laryngeal reflux effects the reflux caused by strong acid, while the treatment effect of weak acid and acid-free (pepsin) reflux is poor, which is related to the difference between the mechanism of damage caused by weak acid and pepsin to the throat and the mechanism of direct damage caused by strong acid. Therefore, this study focused on the effects of weak acid and pepsin on laryngeal injury. Common animal models include pigs, dogs, rabbits, and rats. The vocal cord structure of rabbits is the same as that of humans, and rabbits are silent animals, which can avoid the influence of vocal cord movement injury on the results. Therefore, we selected rabbits for modeling.

2. Abstract

Since the main component of stomach acid is hydrochloric acid, to simulate stomach acid, hydrochloric acid was used in this study. Hematoxylin and eosin were used to observe the tissue inflammatory response. The expression of some sentences was modified.

3. Introduction

Revised the wording in this section and added additional references.

4. Methodology

Since there is no specific rabbit laryngoscopy scoring standard, we also used the human laryngoscopy scoring standard previously reported (Belafsky et al.). Air embolization is necessary for rabbit tissue sampling and does not affect the experimental results. Lanthanum nitrate staining is one of the special staining methods for electron microscopy. When the mucosal barrier is intact, lanthanum nitrate does not penetrate the intercellular space; however, if the mucosal barrier is damaged, lanthanum staining can penetrate the intercellular space and inside the cell when the cell dies. DIS is the expansion of the cell gap; that is, when the mucosal barrier is normally intact, the cell connection is tight; however when broken, the cell gap can be observed under the electron microscope. Finally, the expression of E-Cadherin in cells was observed using immunohistochemical staining of arytenoid mucosa. Some background information regarding instantaneous impedance was added to the revised. And new references have been added.

Best regards,
