# Supplementary material for: Weak acid and pepsin reflux induce laryngopharyngeal mucosal barrier injury: A rabbit-model-based study
Source: PLoS One. 2025 Jan 24;20(1):e0315083. doi: 10.1371/journal.pone.0315083 (PMC11759997; doi:10.1371/journal.pone.0315083)
Supplement: Attachment — Submitted filename: Response to Reviewers.docx [file pone.0315083.s004.docx]

**Response to Reviewers**

**Subject: Response to Reviewer #1’s Comments on Manuscript "Weak Acid and Pepsin Reflux Induce Laryngopharyngeal Mucosal Barrier Injury: A Rabbit-Model-Based Study"**

Dear Dr. Flemban,

Thank you very much for your thoughtful suggestions and constructive feedback on our manuscript, "Weak Acid and Pepsin Reflux Induce Laryngopharyngeal Mucosal Barrier Injury: A Rabbit-Model-Based Study." We truly appreciate the time and effort you have dedicated to reviewing our work.

In response to your insightful comments regarding the E-cadherin staining images, we have chosen to upload new images that we believe more accurately reflect our experimental results.

We greatly value your recommendations on utilizing markers such as Occludin, ZO-1, and Vimentin in our future studies. E-cadherin, as you pointed out, is a specific adhesion molecule for epithelial cells, and its downregulation indeed signifies a loss of epithelial characteristics, thereby providing a clear indication of EMT occurrence. We also acknowledge that Occludin, ZO-1, and Vimentin can offer complementary insights that would enhance our understanding of the EMT process. Unfortunately, due to time constraints, we were unable to conduct additional experiments at this stage. We are committed to considering your suggestions in our ongoing research.

Thank you once again for your invaluable input and guidance.

Warm regards,

Ting Chen

**Subject: Response to Reviewer #2’s Comments on Manuscript "Weak Acid and Pepsin Reflux Induce Laryngopharyngeal Mucosal Barrier Injury: A Rabbit-Model-Based Study"**

Dear Dr. Adedokun,

Thank you very much for your valuable feedback and constructive suggestions regarding our manuscript, "Weak Acid and Pepsin Reflux Induce Laryngopharyngeal Mucosal Barrier Injury: A Rabbit-Model-Based Study."

We appreciate your observation about the clarity and strength of our response compared to the original document. In light of your feedback, we have revised the introduction to more clearly highlight the contribution of our study to the existing knowledge on weak acids and their implications for treatment. We have incorporated additional information and relevant references to provide a stronger foundation and underscore the importance of our findings.

Regarding the title capitalization, we have ensured that it adheres to the proper formatting guidelines. We understand the importance of these details and have taken care to address them appropriately in the revised manuscript.

Thank you once again for your insightful comments, which have significantly contributed to improving our manuscript.

Sincerely,

Ting Chen
